# Development of a Rapid Throughput Assay for Identification of hNa_v_1.7 Antagonist Using Unique Efficacious Sodium Channel Agonist, Antillatoxin

**DOI:** 10.3390/md14020036

**Published:** 2016-02-16

**Authors:** Fang Zhao, Xichun Li, Liang Jin, Fan Zhang, Masayuki Inoue, Boyang Yu, Zhengyu Cao

**Affiliations:** 1 State Key Laboratory of Natural Medicines, China Pharmaceutical University, Nanjing 211198, China; zhaofang0927@yahoo.com (F.Z.); xichun_li@163.com (X.L.); liangjin1975@cpu.edu.cn (L.J.); boyangyucpu@163.com (B.Y.); 2 Jiangsu Provincial Key laboratory for TCM Evaluation and Translational Development, China Pharmaceutical University, Nanjing 211198, China; 3 School of Life Science and Technology, China Pharmaceutical University, Nanjing 211198, China; 4 Graduate School of Pharmaceutical Sciences, The University of Tokyo, Tokyo 113-0033, Japan; inoue@mol.f.u-tokyo.ac.jp

**Keywords:** antillatoxin, FMPblue, membrane potential, hNa_v_1.7, rapid throughput

## Abstract

Voltage-gated sodium channels (VGSCs) are responsible for the generation of the action potential. Among nine classified VGSC subtypes (Na_v_1.1–Na_v_1.9), Na_v_1.7 is primarily expressed in the sensory neurons, contributing to the nociception transmission. Therefore Na_v_1.7 becomes a promising target for analgesic drug development. In this study, we compared the influence of an array of VGSC agonists including veratridine, BmK NT1, brevetoxin-2, deltamethrin and antillatoxin (ATX) on membrane depolarization which was detected by Fluorescence Imaging Plate Reader (FLIPR) membrane potential (FMP) blue dye. In HEK-293 cells heterologously expressing hNa_v_1.7 α-subunit, ATX produced a robust membrane depolarization with an EC_50_ value of 7.8 ± 2.9 nM whereas veratridine, BmK NT1, and deltamethrin produced marginal response. Brevetoxin-2 was without effect on membrane potential change. The ATX response was completely inhibited by tetrodotoxin suggesting that the ATX response was solely derived from hNa_v_1.7 activation, which was consistent with the results where ATX produced a negligible response in null HEK-293 cells. Six VGSC antagonists including lidocaine, lamotrigine, phenytoin, carbamazepine, riluzole, and 2-amino-6-trifluoromethylthiobenzothiazole all concentration-dependently inhibited ATX response with IC_50_ values comparable to that reported from patch-clamp experiments. Considered together, we demonstrate that ATX is a unique efficacious hNa_v_1.7 activator which offers a useful probe to develop a rapid throughput screening assay to identify hNa_v_1.7 antagonists.

## 1. Introduction

Voltage-gated sodium channels (VGSCs) are responsible for the rising phase of the action potential in excitable cells such as neurons, cardiac myocytes and skeletal muscle myocytes [1,2]. VGSCs are composed of voltage-sensing and pore-forming elements in one principal α-subunit and one or two auxiliary β-subunits which alter the channel physiological properties and subcellular localization [3]. Based on the amino acid similarity of the α-subunit isoforms, nine VGSC subtypes have been described termed Na_v_1.1–Na_v_1.9 [4].

VGSCs represent the molecular targets for a broad range of potent neurotoxins that bind to at least six distinct neurotoxin sites on the sodium channel α-subunit and affect the ion permeation and gating of sodium channels [3]. These toxins include tetrodotoxin (TTX), saxitoxin, and μ-conotoxin (site 1); lipid-soluble alkaloid toxins, including batrachotoxin, veratridine, aconitine, and grayanotoxin (site 2); polypeptide sea anemone and α-scorpion toxins (site 3); β-scorpion toxins (site 4); marine toxins such as brevetoxins (PbTxs) and ciguatoxins (site 5); and δ-conotoxins (site 6) [5]. In addition, pyrethroid insecticides act at a distinctive site on the sodium channel α-subunit to enhance channel activity by shifting activation to more negative membrane potentials as well as by delaying inactivation [6].

The expression of sodium channel α-subunits is tissue-dependent. Na_v_1.7 is preferentially expressed in the nociceptive neurons such as dorsal root ganglion and trigeminal ganglion as well as the sympathetic ganglion neurons [7] producing “threshold currents” close to resting potential, amplifying small depolarization such as generator potentials [8]. The role of Na_v_1.7 in the nociception and pain has been well established [9]. Several gain-of-function mutations in *SCN9A* which encodes Na_v_1.7 caused primary erythromelalgia, resulting in burning pain and flushing [10,11]. On the contrary, the inflammatory pain responses were reduced or abolished in nociceptor-specific Na_v_1.7 knock-out mice [12,13]. Deleting *SCN9A* in both sensory and sympathetic neurons abolished the pain sensations and recapitulated the pain-free phenotype seen in humans with *SCN9A* loss-of-function mutations [13]. These observations highlighted Na_v_1.7 as a potentially useful target for the development of novel analgesics.

Patch clamp electrophysiology is the gold standard for characterizing compound activity on the ion channels. While irreplaceable to study the millisecond kinetics of activation and inactivation of VGSCs, the patch clamp technique is laborious and extremely slow, which has greatly limited the utility of this technique to discover novel chemotypes targeting on ion channels. Recently, the automated, multichannel, voltage-clamp instruments provided the possibility for screening larger libraries of compounds. Unfortunately, automated electrophysiology is still quite expensive and is available in few academic laboratories [14,15].

Thus, higher-throughput, less expensive techniques are valuable alternatives to automated electrophysiology. Recently, researchers have developed several fluorescence-based rapid throughput assays for ion channel ligands discovery. The high throughput screening (HTS) thallium (Tl^+^)-flux assay has been developed to discover modifiers of K^+^ channels [16,17,18], K^+^-coupled chloride cotransporters [19], and Na^+^ and K^+^-coupled chloride cotransporters [20]. Many efforts have been made to develop the functional HTS assays to identify the VGSC modifiers. These assays including using sodium specific fluorescence dye, sodium-binding benzofuran isophthalate/acetoxymethyl ester (SBFI/AM) in neurons [5,21,22] and in heterologously expressed VGSCs cells [23]. However, the sodium bounded SBFI/AM fluorescence required excitations at two wavelengths (340 and 380 nm) which limited the throughput. In addition, in a heterologously expressed system, the fluorescence signal to noise ratio of the sodium channel agonists at defined recognition sites was minimal [23]. Membrane potential dye such as DiSBAC2(3) was also used to develop the HTS assay for discovering the VGSC antagonists [24,25]. In addition to its two wavelength recording (460 nm and 580 nm), the robust FRET fluorescence signals only can be achieved by co-application of two sodium channel agonists simultaneously. This co-application of two agonists resulted in a low Z′ value (0.15–0.45) which was not suitable for HTS assay [25]. Therefore, an agonist which can efficaciously activate the VGSCs is needed. Antillatoxin (ATX), a structurally unique lipopeptide produced by the marine cyanobacterium, *Lyngbya majuscula*, is a VGSC agonist [5,23,26,27]. ATX binds to a topological distinct neurotoxin site and allosterically potentiates the VGSC agonists-induced [^3^H]batrachotoxin binding [27]. More importantly, functional analysis has demonstrated that ATX displays the highest efficacy on the stimulation of sodium influx compared to other VGSC agonists [5,23].

In this study, we compared the influence of a range of VGSC agonists which bound to VGSC distinct neurotoxin sites using FLIPR membrane potential (FMP) blue dye in a 96-well format in hNa_v_1.7-HEK-293 cells. Among the agonists tested, only ATX produced a potent and efficacious membrane depolarization, providing a good signal/noise ratio. We further demonstrated that six VGSC antagonists including lidocaine, lamotrigine, phenytoin, carbamazepine, riluzole, and 2-amino-6-trifluoromethylthiobenzothiazole (SKA-19) all concentration-dependently inhibited ATX response with IC_50_ values comparable to that reported from patch-clamp experiments. These data suggested that ATX might represent a useful probe for developing an HTS assay to identify Na_v_1.7 antagonists.

## 2. Results

### 2.1. Influence of VGSC Agonists on Membrane Depolarization in HEK-293 Cells Stably Expressing hNa_v_1.7

**Figure 1:**
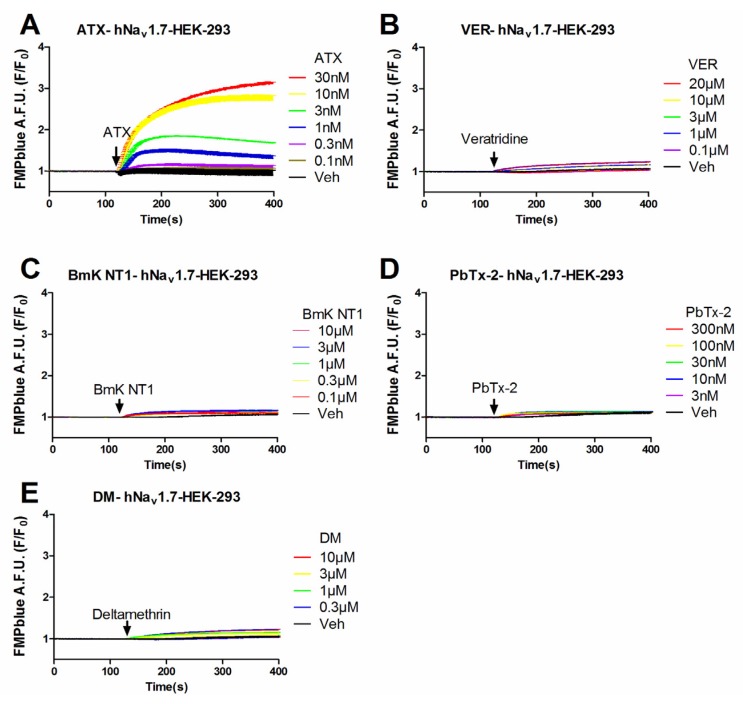
Time-response relationships for ATX (**A**); veratridine (VER) (**B**); BmK NT1 (**C**); PbTx-2 (**D**); and deltamethrin (DM) (**E**) on membrane depolarization in hNa_v_1.7-HEK-293 cells. This experiment was performed in three independent cultures, each in triplicate.

**Figure 2:**
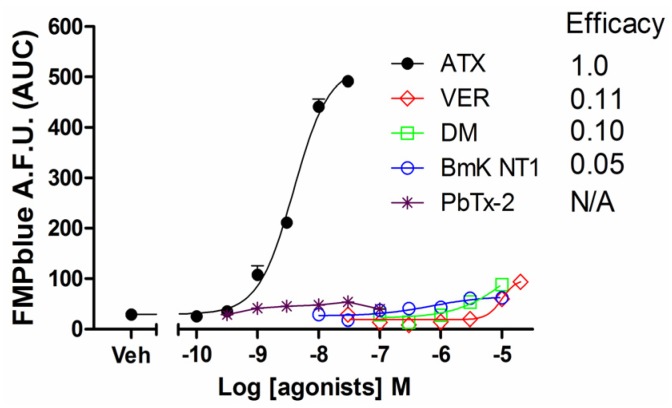
Concentration-response relationship curves for ATX, veratridine, deltamethrin, BmK NT1 and PbTx-2 induced membrane potential changes. Each data point represents the mean ± SEM from two experiments, each in triplicates. ATX produced an efficacious response in the membrane depolarization while veratridine, deltamethrin and BmK NT1 only produced marginal response with efficacies of 0.11, 0.10, and 0.05 respectively. PbTx-2 was without effect on the membrane depolarization.

Previous studies have demonstrated that VGSC agonists such as veratridine, brevetoxin-2 (PbTx-2) had minimal effects on the stimulation of sodium influx or membrane depolarization in HEK-293 cells stably expressing VGSCs [23,25]. To identify an efficacious VGSC agonist which can provide a good signal/noise ratio, we examined the response on membrane depolarization in hNa_v_1.7 expressed HEK-293 cells of five VGSC agonists which bound to topologically distinct neurotoxin sites including veratridine (VER, neurotoxin site 2), BmK NT1 (a site 3 α-scorpion toxin) [22], PbTx-2 (neurotoxin site 5) [5], deltamethrin (unrecognized neurotoxin site) [21], and ATX (unrecognized neurotoxin site) [23]. ATX produced a robust membrane depolarization in a concentration-dependent manner (Figure 1A). Veratridine (up to 20 μM), BmK NT1 (up to 10 μM) and deltamethrin (up to 10 μM) produced a marginal response on the membrane depolarization (Figure 1B,C,E). The neurotoxin site 5 agonist PbTx-2 was without effect on the membrane potential change in hNa_v_1.7-HEK-293 cells (Figure 1D). The EC_50_ value for ATX-stimulated membrane depolarization (area under curve, AUC *vs* Log (concentration)) was 7.8 ± 2.9 nM with a maximal response of 11.7-fold of vehicle control (Figure 2). Compared to the maximal ATX response (efficacy defined as 1), the maximal responses of veratridine, deltamethrin and BmK NT1 were only 0.11, 0.10 and 0.05, respectively (Figure 2).

### 2.2. ATX-Induced Membrane Depolarization Was Dependent on the Activation of hNa_v_1.7

**Figure 3:**
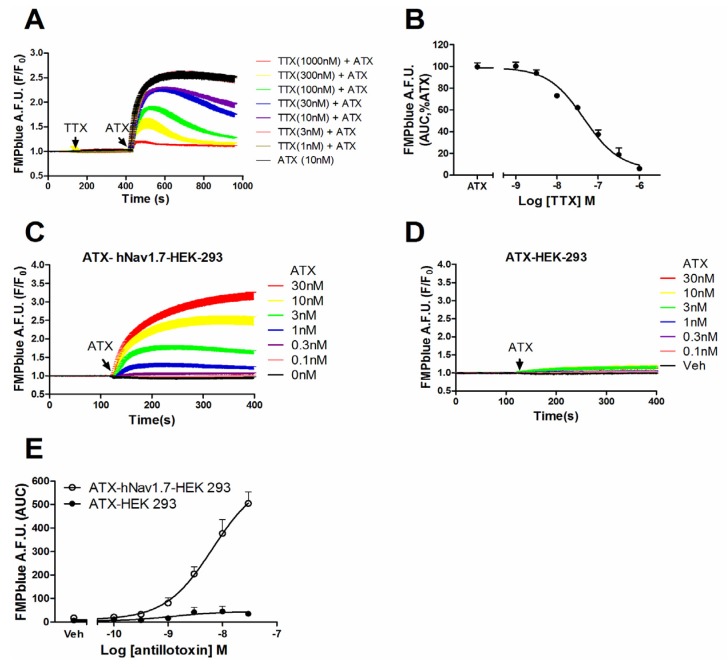
ATX-produced membrane depolarization was dependent on the activation of hNa_v_1.7. (**A**) TTX antagonized ATX-induced membrane depolarization in hNa_v_1.7 HEK-293 cells as a function of time; (**B**) Concentration-response curve for TTX suppressed ATX-induced depolarization in hNa_v_1.7 HEK 293 cells. Each data point represents the mean ± SEM from two independent cultures performed in triplicate; (**C**) and (**D**) ATX response on membrane depolarization in hNa_v_1.7-HEK-293 cells and null HEK-293 cells as a function of time, respectively; (**E**) Concentration-response relationships of ATX response on membrane depolarization in hNa_v_1.7-HEK-293 cells and null HEK-293 cells. This experiment was performed in two independent cultures, each in triplicate with similar results.

Given the efficacious response on the membrane potential change in hNa_v_1.7-HEK-293 cells, we examined whether this membrane depolarization was from the activation of hNa_v_1.7. Pre-treatment of TTX, a pore blocker of VGSC, concentration-dependently suppressed the ATX (10 nM)-induced membrane depolarization with an IC_50_ value of 49.8 nM (31.0–80.4 nM, 95% CI) (Figure 3A,B). In null HEK-293 cells, ATX produced minimal response on the membrane depolarization which, compared to the ATX-induced response in hNa_v_1.7-HEK-293 cells, was marginal (Figure 3C–E).

### 2.3. Z′ Factor Determination

**Figure 4:**
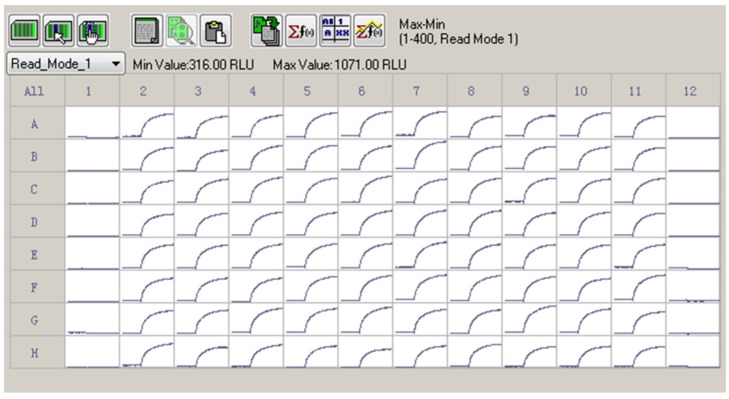
A representative 96-well plate for ATX (30 nM) response on the membrane depolarization. The cells in Columns 2 to 11 were exposed to 30 nM of ATX. The Columns 1 and 12 were negative controls (0.1% DMSO). ATX produced a robust, yet consistent membrane depolarization. The Z′ factor was calculated to be 0.7589. This experiment was performed in two independent cultures.

Given the efficacious ATX response on the membrane depolarization which was solely dependent on the hNa_v_1.7 activation, we determined Z′ factor to test the suitability to use ATX and membrane potential dye for HTS assay. As shown in Figure 4, 30 nM ATX produced a robust, yet consistent response on the membrane depolarization. The calculated Z′ value was 0.7598.

### 2.4. Influence of an Array of VGSC Antagonists on ATX-Induced Membrane Depolarization

**Figure 5:**
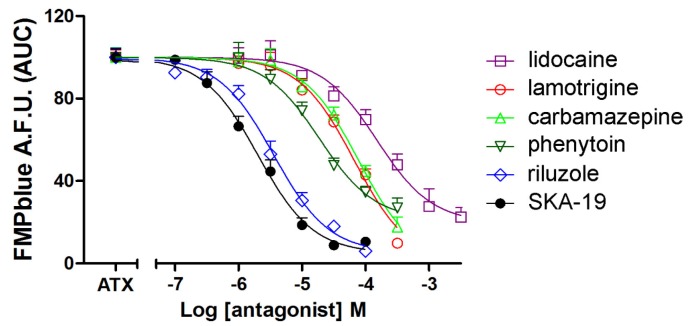
Influence of VGSC antagonists including riluzole, SKA-19, phenytoin, lidocaine, carbamazepine, and lamotrigine on ATX (10 nM)-induced membrane depolarization. Data are presented as percentage of 10 nM ATX-induced fluorescence change. Each data point represents the mean ± SEM from two experiments, each in triplicates.

We next tested the influence of six VGSC antagonists on ATX (10 nM)-induced membrane depolarization. All the VGSC antagonists tested including lidocaine, lamotrigine, phenytoin, carbamazepine, riluzole, and SKA-19 produced concentration-dependent inhibition of ATX (10 nM)-induced membrane depolarization (Figure 5). The IC_50_ values for SKA-19, riluzole, phenytoin, lamotrigine, carbamazepine, and lidocaine were 2.02 (1.49–2.74 μM, 95% CI), 3.58 (2.67–4.80 μM, 95% CI), 18.7 (11.8–29.7 μM, 95% CI), 66.3 (40.7–108.1 μM, 95% CI), 77.7 (49.9–121.0 μM, 95% CI) and 150.6 (92.9–244.0 μM, 95% CI), respectively (Table 1). The IC_50_ values generated here are consistent to that generated from patch clamp (Table 1). It should be noted that riluzole, SKA-19, carbamazepine, and lamotrigine all produced nearly complete inhibition on ATX-induced membrane depolarization. However, the maximal inhibition of lidocaine and phenytoin on ATX-induced depolarization was somewhat smaller representing a maximal suppressing of 80.2% ± 5.8% and 78.8% ± 5.5%, respectively.

**Table 1:** Comparison of the IC_50_ values generated from this study with that from patch clamp.

Compounds	IC_50_ (μM) (95% CI)	Patch-Clamp IC_50_ (μM)	Reference
SKA-19	2.02 (1.49–4.74)	5.8	[28]
Riluzole	3.58 (2.67–4.80)	2	[29]
Phenytoin	18.7 (11.8–29.7)	31.6	[24]
Lamotrigine	66.3 (40.6–108.1)	79	[30]
Carbamazepine	77.7 (49.9–121.0)	101	[30]
Lidocaine	150.6 (92.9–244.0)	110	[31]

## 3. Discussion

In primary cultured neuronal preparation, the VGSC agonists ATX, veratridine, BmK NT1, PbTx-2 and deltamethrin which bound to topologically distinct neurotoxin sites all produced robust and significant sodium influx with distinct efficacies [5,22,23]. However, several studies have pointed out that in heterologous expression systems, these VGSC agonists produced a minimal response on both sodium influx and membrane potential [5,25]. In this study, we examined the ability of five VGSC agonists which bound to topologically distinctive neurotoxin sites to stimulate the membrane depolarization in hNa_v_1.7-HEK-293 cells. Consistent with the previous studies [23,25], veratridine (neurotoxin site 2) only produced minimal response on the membrane depolarization whereas PbTx-2 (neurotoxin site 5) was without effect in hNa_v_1.7-HEK-293 cells. We further demonstrated that deltamethin, which bound to an undefined neurotoxin site delaying the inactivation of the VGSCs [32], produced minimal response on the membrane potential changes. In addition, a scorpion toxin, BmK NT1 which likely bound to neurotoxin site 3 and prolonged the inactivation of the VGSCs in neurons [22] only produced marginal response to stimulate membrane depolarization. However, ATX produced an efficacious response on the membrane depolarization. Although the sodium channel expression density may partially account for the response discrepancy between neuronal and heterologously expression system [33], the hNa_v_1.7-HEK-293 cells had little β-subunits co-expression. Sodium channel β-subunits regulate α-subunit function at multiple levels including mRNA expression, channel stabilization/trafficking and direct channel modulation [34]. In addition, the resting membrane potential for HEK-293 cells is relatively depolarized (−35 ± 5 mV) [35]. At this depolarized resting membrane potential, most hNa_v_1.7 channels are in inactivated state [35]. Veratridine, BmK NT1, PbTx-2 and deltamethrin were demonstrated to primarily delay the VGSCs inactivation kinetics, but not the activation kinetics [22,32,36], an alternative explanation for these four VGSC agonists only producing marginal response in hNa_v_1.7-HEK-293 cells. Although the detailed electrophysiological characterization of ATX on the VGSC remained to be established, the efficacious response on the membrane depolarization in hNa_v_1.7-HEK-293 cells highly suggested that ATX response was not dependent on the β-subunits. The unique efficacious response of ATX also suggested that ATX may interact preferentially with the inactivated state of VGSC α-subunits.

The ATX response in hNa_v_1.7-HEK-293 cells was from activation of hNa_v_1.7 inasmuch as TTX completely suppressed the ATX-induced membrane depolarization. Furthermore, in null HEK-293 cells, ATX produced marginal membrane depolarization. The marginal membrane depolarization possibly was derived from the endogenously expressed VGSCs in null HEK-293 cells [37]. A rapid throughput assay to identify Na_v_1.7 antagonists has been developed by co-application of veratridine and a scorpion toxin SVqq to achieve the robust fluorescence signals through activating VGSCs. However, this co-application of two agonists resulted in a low Z′ value (0.15–0.45) [25], which was not suitable for the HTS assay. We demonstrated here that ATX produced a robust as well as consistent fluorescence change in a whole 96-well plate with a Z′ value of 0.7589. The Z′ value greater than 0.5 was thought to be suitable for the HTS assay [25].

In this study, we further demonstrated that an array of VGSC antagonists, including SKA-19, riluzole, phenytoin, lamotrigine, carbamazepine, and lidocaine all concentration-dependently suppressed ATX-induced membrane depolarization. It has been demonstrated that the IC_50_ values generated from a fluorescence-based assay is typically five-fold less potent than that generated from patch-clamp [24,30]. However, we demonstrated that the IC_50_ values for these VGSC inhibitors generated in this study were consistent with that from patch-clamp experiments [24,28,29,30,31,38,39] (Table 1). Riluzole has been reported to suppress TTX-sensitive VGSC current in inactivated state with IC_50_ values of 2 μM in dorsal root ganglion neurons, in which Na_v_1.7 was the major TTX-sensitive VGSC subtype. At resting state, riluzole displayed a much lower affinity with an IC_50_ value of 90 μM, suggesting riluzole preferred to bind to the inactivated state of VGSCs [29]. We demonstrated that riluzole inhibited the ATX-induced membrane potential changes with an IC_50_ value of 3.58 μM which was comparable to its affinity in the inactivated state [29]. SKA-19, a thioanalog of riluzole, is a use- and state-dependent VGSC antagonist with IC_50_ value of 5.8 ± 2.6 μM. We demonstrated here that SKA-19 suppressed the ATX (10 nM)-induced membrane depolarization in hNa_v_1.7-HEK-293 cells with an IC_50_ value of 2.02 μM, a value similar to that from voltage-patch clamp experiment [28]. The anticonvulsants phenytoin, lamotrigine, and carbamazepine were also preferred to bind to inactivated state of VGSCs [40,41,42,43,44,45,46,47]. The affinities for these anticonvulsants on the inactivated state are much higher than that on the closed and open states [48]. We demonstrated that the IC_50_ values for lamotrigine, carbamazepine and phenytoin suppression of ATX-induced membrane depolarization in hNa_v_1.7-HEK-293 cells were 66.3, 77.7 and 18.7 μM, respectively, which were more consistent with their affinities on the inactivated state. The local anesthetics, lidocaine has been reported to affect the steady-state fast inactivation of Na_v_1.7 channels with an IC_50_ value of 110 ± 20 μM [31] which was also consistent with current finding (150.6 μM, 92.9–244.0 μM, 95% CI). Considered together, it appeared that the IC_50_ values generated from current fluorescence-based HTS assay were more consistent with their respective affinity in the inactivated state.

It has been reported that riluzole, lidocaine, phenytoin bound to distinct sites of the sodium channels [49,50,51,52]. For example, riluzole interacts with amino acids residues TYR 1787, LEU 1843 and GLN 1799 located in the transmembrane segment S6 of domain IV of the α-subunit [53]. Lidocaine binds to the local anesthetics site located in the channel pore [39,54]. Phenytoin binds to the S6 segments of domains III and IV of the Na^+^ channel α-subunit [40,47,55,56]. In addition, TTX binds to another neurotoxin site distinct to those use- and steady-state blockers. The comparable IC_50_ values between current study and reported previously suggesting that the fluorescence-based assay developed here was capable of identifying the hNa_v_1.7 inhibitors bound to distinct neurotoxin sites on the α-subunits of VGSCs.

It has been reported that fluorescence-based assays are often subject to high false positive hits [57,58]. Further study was required to screen a chemical library to determine the liability of the assay. Nevertheless, the HTS assay developed here may represent a useful alternative for the primary screen to identify hNa_v_1.7 antagonists with novel pharmacophores.

## 4. Materials and Methods

### 4.1. Materials

FMP blue dye was obtained from Molecular Devices (Sunnyvale, CA, USA). ATX was synthesized as described previously and was characterized to be above 95% purity [59]. G-418, penicillin, streptomycin, heat inactivated fetal bovine serum, poly-d-lysine (molecular weight >300,000), riluzole, veratridine and carbamazepine, deltamethrin, PbTx-2, TTX, and lamotrigine were obtained from Sigma-Aldrich (St. louis, MO, USA). SKA-19 was provided by Prof. Wulff at the University of California, Davis as described previously [28] and was characterized to be greater than 95% purity. Lidocaine was purchased from Abcam (Cambridge, MA, USA). The HEK-293 stably expressed Na_v_1.7 was a generous gift from Dr. Lossin (University of California, Davis) and was the same line used as described previously [28].

### 4.2. Cell Culture

Human Embryonic Kidney 293 (HEK-293) cells were cultured in DMEM with glutamine supplemented with 10% fetal bovine serum (FBS), 100 units/mL penicillin and 0.1 mg/mL streptomycin. HEK-293 cells stably expressed hNa_v_1.7 were cultured in DMEM supplemented with 10% FBS, 100 units/mL penicillin, 0.1 mg/mL streptomycin, and 500 μg/mL G-418. All cells were grown routinely as monolayers on poly-d-lysine coated T-75 flask in an atmosphere of 5% CO_2_ and 95% humidity at 37 °C.

### 4.3. Membrane Potential Change Detection

Membrane potential changes in HEK-293 or HEK-293 stably expressed hNa_v_1.7 were determined using the FMP blue dye (Molecular Devices, Sunnyvale, CA, USA). The cells were plated onto poly-d-lysine (10 μg/mL) coated, 96-well, black-walled, clear-bottom plates at an initial density of 20,000 cells/well and cultured for 6 h. Cells were removed of their medium and 150 μL of 1× dye solution (1 bottle dissolved in 50 mL Locke’s buffer, in mM: 8.6 HEPES, 5.6 KCl, 154 NaCl, 5.6 Glucose, 1.0 MgCl_2_, 2.3 CaCl_2_, 0.0001 glycine, pH 7.4) was added to each well. Cells were then incubated at room temperature for 30 min. The plate was then transferred to a FLIPR^®TETRA^ (Molecular Devices, Sunnyvale, CA, USA) chamber. Cells were excited at 510–545 nm and emission at 565–625 nm was recorded at 1 s intervals. After recording the basal fluorescence for 120 s, 50 μL of sodium channel agonists at different concentrations (prepared in 1× dye at 4× final drug concentrations) or vehicle (0.4% DMSO, 4×) were added to different wells by an automated, programmable pipetting system. The fluorescence was recorded for additional 5–6 min at a sampling rate of 1 s. To examine the VGSC antagonist response on ATX-stimulated membrane depolarization, after recording the basal fluorescence for 120 s, different concentrations of VGSC antagonists were added to corresponding wells and the fluorescence was recorded for additional 5 min followed by an addition of 40 nM (4×, final concentration, 10 nM) ATX. The fluorescence signals were presented as F/F_0_, where F was defined as the fluorescence at different time points; F_0_ was the basal fluorescence averaged from initial 5 data points.

### 4.4. Data Analysis

Time-response and concentration-response relationships curves were generated using GraphPad Prism 5 software (GraphPad Software, Inc., San Diego, CA, USA). The EC_50_ value for VGSC agonists-induced membrane depolarization was determined by non-linear regression analysis using a logistic equation. The IC_50_ values of VGSC antagonists against ATX-induced membrane depolarization was determined by non-linear regression analysis using a logistic equation. Z′ factor was calculated using the following equation as described previously [60]: Z′ = 1 − [3 SD of sample + 3 SD of control]/[mean of sample − mean of control]. Each experiment was repeated at least twice in independent cultures performed at least in triplicate.

## 5. Conclusions

The current study investigated an array of VGSC agonists to stimulate the membrane depolarization in hNa_v_1.7-HEK-293 cells. We demonstrated that ATX but not other VGSC agonists tested produced efficacious response on the membrane depolarization. We further demonstrated that ATX can serve as a probe to develop an HTS assay for identifying hNa_v_1.7 antagonist with distinct binding sites.

## Abbreviations

ATX: antillatoxin
FBS: fetal bovine serum
FLIPR: fluorescence imaging plate reader
FMP: FLIPR membrane potential
HEK: human embryonic kidney
hNa_v_1.7: human voltage-gated sodium channel subtype 1.7
HTS: high throughput screen
PbTxs: brevetoxins
SBFI/AM: sodium-binding benzofuran isophthalate/acetoxymethyl ester
SKA-19: 2-Amino-6-trifluoromethylthiobenzothiazole
Tl^+^: thallium
TTX: tetrodotoxin
VGSCs: voltage-gated sodium channels
